# Contingencies of Self-Worth on Positive and Negative Events and Their Relationships to Depression

**DOI:** 10.3389/fpsyg.2018.02372

**Published:** 2018-12-03

**Authors:** Cheng-Hong Liu, Po-Sheng Huang

**Affiliations:** ^1^Department of Educational Psychology and Counseling, National Tsing Hua University, Hsinchu, Taiwan; ^2^Graduate Institute of Digital Learning and Education, National Taiwan University of Science and Technology, Taipei, Taiwan

**Keywords:** contingencies of self-worth, positive contingencies of self-worth, negative contingencies of self-worth, contingent self-esteem, depression

## Abstract

Previous studies have found that the relationship between contingencies of self-worth (CSW) and depression was generally weak. We posited that this is partly because previous studies assumed CSW on positive and negative events as the same construct (one-dimensional CSW), which should be better conceptualized as two distinctive dimensions (two-dimensional CSW) in explaining depression. A total of 393 undergraduates from Taiwan completed the measures regarding one-dimensional CSW, two-dimensional CSW, and depression. After dividing CSW of seven classic domains into two dimensions of positive and negative CSW, the results of confirmatory factor analyses showed that the two-dimensional CSW model had better model fit than the one-dimensional model in all domains. Furthermore, relative to using one-dimensional CSW as a predictor of depression, the variance accounted for largely increased when positive and negative CSW entered simultaneously in the regression equation. The results suggest that CSW on positive and negative events should be seen as two dimensions and this perspective may largely increase the explanatory power of CSW in explaining mental health.

## Introduction

Previous studies indicated that individuals not only differ in the typical or trait level of self-esteem but also in their contingencies of self-worth (CSW), which determines how much their self-worth would fluctuate around the typical level of self-esteem in response to positive and negative events (Crocker and Wolfe, 2001). For a person whose self-worth is more contingent or dependent on a specific domain, negative events in this domain should result in more drops and positive events should result in more increases in self-esteem (Crocker et al., 2003). To date, a number of CSW domains and measures have been identified and developed. For example, Crocker et al. (2003) identified and developed measures to assess five external (i.e., others' approval, competition, academics, appearance, family support) and two internal (i.e., virtue, God's love) sources of self-esteem. Many other CSW domains and measures have also been developed according to this classical study, such as friendship contingent self-esteem (Cambron et al., 2010), relationship contingent self-esteem (Knee et al., 2008), and workplace performance contingent self-esteem (Ferris et al., 2010).

Depression is one of the most common mental health problems worldwide. Numerous studies in psychology have attempted to identify risk factors that affect depression. Among the previous CSW studies, one direction of research has also been aimed at an understanding of the relationship between CSW and depression. For example, Sargent et al. (2006) examined the relationship between CSW and vulnerability to depression over the first semester of college in a sample of college freshmen. They found that college freshmen with higher levels of external CSW (in a composite measure of four external CSW domains which were others' approval, appearance, competition, academics) showed more increases in depressive symptoms over the first semester of college. However, internal CSW (i.e., God's love, virtue) was not associated with the level of depressive symptoms. Burwell and Shirk (2006) also found that adolescents' CSW (in a composite measure of four external CSW domains which were social acceptance and approval, academic performance, activity performance, and physical appearance) was associated with increases in their depressive symptoms, implicating that CSW is an important risk factor to depressive symptoms during adolescence. In addition, Sanchez and Crocker (2005) revealed that external CSW (including others' approval, appearance, competition, academics domains) showed a positive correlation with depression. Several other studies also showed that the extent to which individuals' self-esteem was contingent on a specific external CSW domain such as friendship (Cambron et al., 2010), academics (Schöne et al., 2015), and appearance (Schwinger et al., 2017) was associated with greater depressive symptoms. These studies demonstrated that internal CSW (e.g., virtue and God's love) may be unrelated or even negatively related to depression (see also Crocker et al., 2003). However, having self-worth that is highly dependent on a particular external domain (e.g., academics, appearance, others' approval, competition, and friendship), which would lead to more self-esteem instability (greater fluctuations in self-esteem), is associated with greater depressive symptoms (Crocker and Park, 2004; Crocker and Knight, 2005; Cambron et al., 2010; Wouters et al., 2013a).

However, it's noteworthy that although CSW did generally associate with depression in the expected ways in the above-mentioned studies, the relationship seemed to be generally weak. Whether these studies used a cross-sectional design or a longitudinal design, the proportion of variance in depression accounted for by CSW was generally <10% (see also Crocker et al., 2003, p. 905). Some studies even indicated that CSW did not predict higher levels of depressive symptoms when controlling for self-esteem (Wouters et al., 2013b; Sowislo et al., 2014). Why is the relationship between CSW and depression generally weak? We posited that this may be partly because previous studies assumed CSW on positive and negative events as the same construct (one-dimensional CSW), which may be better conceptualized as two distinctive dimensions (two-dimensional CSW) in explaining depression. The following sections introduce the related concepts.

### CSW on positive and negative events as the same construct (one-dimensional CSW)

Crocker et al. (2003) tested the structure of CSW by using a self-developed CSW scale. In this scale, five items assessed whether self-esteem depends on outcomes in each of seven domains (others' approval, competition, academics, appearance, family support, virtue, and God's love). Three types of items were included: (1) “up” items indicating that self-esteem increases in response to positive outcomes; (2) “down” items indicating that self-esteem decreases in response to negative outcomes; and (3) “depends” items indicating that self-esteem depends on outcomes in the domain without specifying whether the outcomes are positive or negative. Crocker et al. (2003) used 1418 college students' data to conduct confirmatory factor analyses (CFAs) to compare the plausibility of various theoretical models. They found that among these models, the correlated seven-factor model that adopts the seven CSW domains as seven latent factors and all the items belonging to each CSW domain (including three types of items) as the measurement indicators had the best goodness of fit, confirming that CSW can be divided into seven domains.

Among the competing models, Crocker et al. (2003) tested a correlated three-factor model in which using the three types of “up,” “down,” and “depends” items as three latent factors and items of seven CSW domains as measurement indicators (e.g., for the “up” latent factor, all the “up” items from the seven CSW domains were all measurement indicators). The results showed that the model fit was low, indicating that CSW cannot be divided into these three factors. Thus, the following researchers also tended to perceive CSW on positive and negative events as the same one-dimensional concept. When exploring issues regarding CSW, most researchers tended to sum the scores of participants for the three types of items, calculated the mean score for a specific CSW domain, and conducted the following analyses (e.g., Sanchez and Crocker, 2005; Sargent et al., 2006; Cambron et al., 2010; Ferris et al., 2010; Rouse, 2011; Schwinger et al., 2017).

### CSW on positive and negative events as distinctive constructs (two-dimensional CSW)

Although most researchers perceive CSW on positive and negative events as the same one-dimensional concept, we considered that CSW on positive events (positive CSW; PCSW) and CSW on negative events (negative CSW; NCSW) may be better conceptualized as two different dimensions for the following reasons. First, it seems not appropriate to conclude that PCSW and NCSW belong to the same one-dimensional concept based on the above-mentioned poor correlated three-factor model. This is because each factor in the model comprised measurement indicators (items) from the seven CSW domains. Among these seven CSW domains, several domains involved disparate concepts (e.g., academics vs. God's love); thus, achieving a poor goodness of fit when employing items from heterogeneous domains as measurement indicators under the same latent factor is understandable. In addition, the correlated three-factor model included the “up,” “down,” and “depends” latent factors, which seemed to overlap with each other. The “up” items measure the CSW of people in response to positive events and the “down” items measure the CSW in response to negative events. However, the “depends” items measure the overall CSW in response to events without specifying whether the outcomes are positive or negative. Therefore, it is possible that the correlations between the “depends” latent factor and the other two latent factors in the model were too strong to result in a good model fit.

In addition, some studies regarding self-esteem also supported the notion that PCSW and NCSW are possibly two distinct dimensions. For example, Vonk and Smit (2012) indicated that CSW measured with positively phrased items (“up” items) and with negatively phrased items (“down” items) are correlated, but they are not the same. In addition, numerous studies have also indicated that the effects of positive and negative events on self-esteem demonstrate various influential mechanisms and effects (e.g., Showers and Zeigler-Hill, 2007; Zell and Alick, 2010; McConnell, 2011). Therefore, the level of self-esteem increase resulting from a positive event may not equal to the level of self-esteem decrease caused by a negative event for an individual. Blaine and Crocker (1993) also indicated that relatively high self-esteem (HSE) people make more self-serving attributions for performance outcomes than relatively low self-esteem (LSE) people. This implies that relatively HSE people may feel good about themselves when they succeed but may not feel bad about themselves when they fail (Dutton and Brown, 1997). However, relatively LSE people possess low self-confidence and greater negative evaluations, which hinder their acceptance toward positive feedbacks but cause them to be easily affected by failures and negative life events. Thus, their self-evaluation may not increase easily during a positive event but may drastically decrease after a negative event (e.g., Dodgson and Wood, 1998). These studies suggest that CSW on positive and negative events are possibly different concepts and may be better conceptualized as two distinctive dimensions.

#### The relationships between PCSW, NCSW, and depression

Many scales implemented by the previous studies to assess CSW contained both the “up” and “down” items and showed at least acceptable internal consistency (e.g., Crocker et al., 2003; Cambron et al., 2010). This suggests that the relationship between PCSW and NCSW should be positive (see also Vonk and Smit, 2012). However, as mentioned above, because PCSW and NCSW are better seen as two distinct dimensions, the positive correlation between them should be moderate. Next, we considered that PCSW may be negatively related to depressive symptoms. Specifically, because people with high PCSW are typically attentive or feel strongly toward positive events, they tend to feel good about themselves (their self-worth is easily increased) and generate positive emotions during positive events (Kernis, 2003; Fredrickson and Losada, 2005). Clinical studies also found that people who are able to accept and use positive and rewarding stimuli in everyday life to enhance their emotions are healthier and less likely to experience negative emotions (Gotlib and Joormann, 2010). Thus, people with high PCSW should be less likely to feel depressed.

On the other hand, NCSW may be positively related to depressive symptoms. People with high NCSW are typically attentive or feel strongly toward negative events; therefore, their self-worth is easily reduced and they tend to experience negative emotions because of negative events. Ego threat studies also indicated that people who cannot effectively respond to negative events have relatively poor psychological adaptation, leading them to perceive low level of happiness and develop depression (vanDellen et al., 2011). Similarly, clinical studies have reflected this perspective that people who focus on negative stimuli or ruminate on negative emotions and life events are less likely to withdraw from a negative emotional state, leading to psychological problems such as depression and anxiety (Schultz and Heimberg, 2008; Gotlib and Joormann, 2010). Thus, people with high NCSW are more likely to feel depressed. In summary, we predicted that people whose self-worth is easily increased by positive events (higher PCSW) in various CSW domains (including internal and external domains) are less likely to exhibit depressive symptoms, and those whose self-worth is easily decreased by negative events (higher NCSW) tend to show more depressive symptoms.

### The present study

Previous studies investigating the relationship between CSW and depression tended to assume people's CSW on positive and negative events as the same construct. These studies generally found that CSW (one-dimensional CSW) did associate with depressive symptoms in the expected ways, but the effect was weak. The current study predicted that PCSW and NCSW may be better seen as two dimensions (two-dimensional CSW) and this perspective may largely increase the explanatory power of CSW in explaining depression. To examine this, we first conducted CFA on each of the seven classic CSW domains proposed by Crocker et al. (2003) to determine whether PCSW and NCSW belong to a single dimension or two distinctive dimensions. Next, we examined whether the proportion of variance in depressive symptoms explained by CSW can be largely enhanced after classifying CSW of each domain into positive and negative dimensions (PCSW and NCSW), even when controlling for global self-esteem.

## Method

### Participants and procedure

A total of 393 undergraduates from two public and two private universities in Taiwan received payment and extra course credit for participation. Participants ranged in age from 18 to 62 years (*M* = 21.22, *SD* = 5.76). Of the 389 participants for whom we obtained demographic information, 64% were women (*n* = 253) and 35% were men (*n* = 136). Excluding the six participants of unknown age, no difference in age was found between women (*M* = 21.01, *SD* = 5.46) and men (*M* = 21.37, *SD* = 5.66), *t*_(381)_ = 0.61, *p* > 0.05. In addition to some filler items, participants completed the measures for global self-esteem, domain-general contingent self-esteem, domain-specific contingencies of self-worth, and depressive symptoms sequentially during class. These measures were translated into Chinese before being administered to the participants. Participants failing to complete all the items in each measure were excluded from the analyses. Finally, they were debriefed and thanked for their participation.

### Measures

#### Self-esteem

We included the 10-item Rosenberg Self-Esteem Questionnaire (RSE; Rosenberg, 1965) as a measure of global self-esteem (e.g., On the whole, I am satisfied with myself). Participants were instructed to complete the instrument according to how they typically or generally feel about themselves. Responses were made on scales ranging from 1 (*strongly disagree*) to 7 (*strongly agree*). The RSE is one of the most commonly used and well-validated measure of global self-esteem. The alpha coefficient for this measure was 0.89 (*N* = 387).

#### General contingent self-esteem

For the validation of the domain-specific CSW scales used in the current study, participants were also invited to indicate their tendency to base their self-worth on general external sources by completing Paradise and Kernis's 15-item Contingent Self-Esteem Scale (as cited in Kernis and Goldman, 2006; e.g., When my actions do not live up to my expectations, it makes me feel dissatisfied with myself), using a 7-point Likert-type scale (1 = *Strongly Disagree*, 7 = *Strongly Agree*). Kernis and Goldman (2006) reported that this measure possesses adequate internal and test-retest reliability. After all items were positively keyed, a mean score was computed; higher scores reflect greater contingent self-esteem. The alpha coefficient for this scale was 0.91 (*N* = 386).

#### Contingencies of self-worth

The Contingencies of Self-Worth Scale (Crocker et al., 2003) was used to assess whether self-esteem depends on outcomes in each of seven domains (i.e., others' approval, appearance, competition, academics, family support, virtue, God's love), with five items in each domain. Three types of items were included: “up” items, “down” items and “depends” items. Most items were worded so that “agree” responses indicated more contingent self-esteem, but some reverse-scored items were included on each subscale. Responses to each item were made on a scale from 1 (*Strongly Disagree*) to 7 (*Strongly Agree*). Sample items were: “I don't care what other people think of me” (“depends” item in the others' approval domain), “My self-esteem does not depend on whether or not I feel attractive” (“depends” item in the appearance domain), “Doing better than others gives me a sense of self-respect” (“up” item in the competition domain), “My self-esteem is influenced by my academic performance” (“depends” item in the academics domain), “When I don't feel loved by my family, my self-esteem goes down” (“down” item in the family support domain), “My self-esteem depends on whether or not I follow my moral/ethical principles” (“depends” item in the virtue domain), “My self-esteem goes up when I feel that God loves me” (“up” item in the God's love domain). Cronbach's alphas for the seven CSW domain measures were 0.86, 0.79, 0.86, 0.75, 0.78, 0.84, and 0.92, respectively. Each of the subscales correlated positively with the general contingent self-esteem (*r*s = 0.61, 0.68, 0.64, 0.61, 0.44, 0.23, 0.25, *p*s < 0.05). Although the correlations in the virtual domain (*r* = 0.23) and the God's love domain (*r* = 0.25) are relatively low, overall the validity seemed acceptable. In addition, compared with the findings by Crocker et al. (2003), most of the subscales correlated in the expected direction with global self-esteem, *r*s = −0.36 (*p* < 0.05), −16 (*p* < 0.05), 0.07 (*p* > 0.05), −0.16 (*p* < 0.05), 0.05 (*p* > 0.05), −0.09 (*p* > 0.05), −0.05 (*p* > 0.05).

It is noteworthy that we aimed to use CFA to test whether CSW in each domain belong to a one-dimensional or two-dimensional concept; however, we cannot use the original Crocker et al. (2003) scale to conduct CFA because the solutions for two-factor CFA model can only be obtained when each of the latent factors possess at least two items. As to their subscales, the other's approval domain does not include “up” items; the competition domain does not include “down” items; the appearance domain has only one “up” and “down” items, respectively; academics and family support domains have only one “down” item; and the virtue domain have only one “up” item (see the numbers in parentheses in NI column in Table 1). Therefore, based on the original scale, we added 24 items to ensure that the seven domains possessed two or more “up” and “down” items (see Appendix). Overall, seven to nine items were included for each domain in the final scale, and each domain comprised two or three “up,” “down,” or “depends” items (see the NI column in Table 1, numbers outside parentheses indicate final item numbers containing the original scale and the added items). Cronbach's alphas for the new subscales of the seven CSW domains were 0.85, 0.87, 0.87, 0.88, 0.87, 0.88, and 0.94, respectively. Each of the subscales correlated positively with the general contingent self-esteem (*r*s = 0.68, 0.72, 0.76, 0.65, 0.48, 0.24, 0.24, *p*s < 0.05) and most of the subscales correlated in the expected direction with global self-esteem, *r*s = −0.31 (*p* < 0.05), −17 (*p* < 0.05), −0.20 (*p* < 0.05), −0.20 (*p* < 0.05), −0.03 (*p* > 0.05), −0.05 (*p* > 0.05), −0.08 (*p* > 0.05). More importantly, the seven new CSW subscales were highly consistent with the original Crocker et al.'s seven CSW measures (*r*s = 0.96,0.95,0.84,0.95,0.93,0.97,0.98, *p*s < 0.05).

**Table 1:** Descriptive Statistics, Internal Consistencies (α), and Intercorrelations for All the Measures.

		**1**	**2**	**3**	**4**	**5**	**6**	**7**	**NI**	***N***	***M***	***SD***	**α**
Others' Approval	1. PCSW	1							2(0)	382	5.72	0.95	0.87
	2. NCSW	0.20*	1						3(2)	385	3.77	1.19	0.72
	3. Depends CSW	0.31*	0.77*	1					3(3)	384	4.23	1.32	0.81
	4. Positive and Negative CSW	0.60*	0.91*	0.77*	1				5	385	4.54	0.88	0.68
	5. All CSW	0.49*	0.90*	0.93*	0.95*	1			8	385	4.43	0.98	0.85
	6. Crocker CSW	0.28*	0.89*	0.97*	0.85*	0.96*	1		5	385	4.03	1.22	0.85
	7. Depression	−0.13*	0.33*	0.24*	0.22*	0.25*	0.27*	1	20	384	1.84	0.46	0.90
Appearance	1. PCSW	1							3(1)	383	5.11	1.00	0.87
	2. NCSW	0.34*	1						3(1)	383	3.77	1.20	0.85
	3. Depends CSW	0.44*	0.67*	1					3(3)	384	4.08	1.17	0.77
	4. Positive and Negative CSW	0.78*	0.85*	0.69*	1				6	381	4.44	0.91	0.82
	5. All CSW	0.70*	0.85*	0.88*	0.95*	1			9	380	4.32	0.92	0.87
	6. Crocker CSW	0.59*	0.74*	0.96*	0.82*	0.95*	1		5	385	4.29	0.99	0.79
	7. Depression	−0.14*	0.34*	0.17*	0.15*	0.17*	0.16*	1	–	–	–	–	–
Competition	1. PCSW	1							3(3)	385	5.70	0.89	0.83
	2. NCSW	0.32*	1						3(0)	382	4.13	1.24	0.85
	3. Depends CSW	0.60*	0.66*	1					3(2)	385	4.88	1.04	0.72
	4. Positive and Negative CSW	0.74*	0.88*	0.78*	1				6	382	4.91	0.87	0.80
	5. All CSW	0.73*	0.84*	0.91*	0.97*	1			9	382	4.90	0.88	0.87
	6. Crocker CSW	0.92*	0.44*	0.82*	0.78*	0.84*	1		5	385	5.50	0.89	0.86
	7. Depression	−0.09	0.31*	0.14*	0.18*	0.17*	−0.02	1	–	–	–	–	–
Academics	1. PCSW	1							3(2)	385	5.38	0.92	0.83
	2. NCSW	0.49*	1						3(1)	384	4.13	1.18	0.77
	3. Depends CSW	0.58*	0.75*	1					3(2)	381	4.36	1.18	0.76
	4. Positive and Negative CSW	0.82*	0.90*	0.78*	1				6	384	4.75	0.90	0.82
	5. All CSW	0.77*	0.89*	0.92*	0.97*	1			9	380	4.62	0.94	0.88
	6. Crocker CSW	0.78*	0.79*	0.88*	0.91*	0.95*	01		5	385	4.74	0.92	0.75
	7. Depression	−0.10	0.23*	0.17*	0.10*	0.14*	0.12*	1	–	–	–	–	–
Family Support	1. PCSW	1							3(2)	383	5.77	0.94	0.83
	2. NCSW	0.38*	1						3(1)	381	4.57	1.27	0.80
	3. Depends CSW	0.63*	0.67*	1					3(2)	383	5.01	1.11	0.69
	4. Positive and Negative CSW	0.77*	0.88*	0.78*	1				6	380	5.17	0.92	0.80
	5. All CSW	0.76*	0.85*	0.91*	0.97*	1			9	379	5.12	0.94	0.87
	6. Crocker CSW	0.80*	0.66*	0.90*	0.86*	0.93*	1		5	384	5.37	0.95	0.78
	7. Depression	−0.16*	0.17*	−0.01	0.04	0.02	−0.04	1	–	–	–	–	–
Virtue	1. PCSW	1							2(1)	385	5.21	1.03	0.71
	2. NCSW	0.60*	1						3(3)	385	4.90	1.11	0.82
	3. Depends CSW	0.57*	0.70*	1					3(1)	384	4.91	1.07	0.73
	4. Positive and Negative CSW	0.84*	0.94*	0.73*	1				5	385	5.03	0.97	0.84
	5. All CSW	0.79*	0.91*	0.90*	0.96*	1			8	385	4.98	0.94	0.88
	6. Crocker CSW	0.73*	0.95*	0.81*	0.96*	0.97*	1		5	385	4.96	1.00	0.84
	7. Depression	−0.14*	0.08	−0.04	−0.01*	−0.02	0.01	1	–	–	–	–	–
God's Love	1. PCSW	1							2(2)	384	3.95	1.63	0.97
	2. NCSW	0.65*	1						3(2)	384	3.08	1.35	0.89
	3. Depends CSW	0.66*	0.88*	1					2(1)	383	3.09	1.41	0.87
	4. Positive and Negative CSW	0.89*	0.93*	0.86*	1				5	385	3.43	1.33	0.91
	5. All CSW	0.84*	0.94*	0.93*	0.99*	1			7	384	3.33	1.31	0.94
	6. Crocker CSW	0.90*	0.90*	0.87*	0.99*	0.98*	1		5	385	3.48	1.36	0.92
	7. Depression	0.08	0.06	0.03	0.00	0.01	−0.03	1	–	–	–	–	–

PCSW, Positive CSW obtained from “up” items; NCSW, Negative CSW obtained from “down” items; Depends CSW, One-dimensional CSW obtained from “depends” items; Positive and Negative CSW, One-dimensional CSW obtained from “up” and “down” items; All CSW, One-dimensional CSW obtained from “up”, “down”, and “depends” items; Crocker CSW, One-dimensional CSW obtained from Crocker et al. (2003) items; _NI_, Numbers of items; Numbers in parentheses indicate numbers of Crocker et al. (2003) items;

* *p < 0.05*.

#### Depression

Depression was assessed with the Center for Epidemiologic Studies Depression Scale (CES-D; Radloff, 1977). The CES-D is a frequently used 20-item self-report measure for the assessment of depressive symptoms in non-clinical, subclinical, and clinical populations. Participants were instructed to assess the frequency of their reactions within the preceding 7 days. Responses were measured on a 4-point scale (0 = rarely or none of the time, < 1 day; 1 = some or a little of the time, 1 to 2 days; 2 = occasionally or a moderate amount of time, 3 to 4 days; 3 = most or all of the time, 5 to 7 days). The alpha reliability of the CES-D was 0.91 (*N* = 379).

## Results

### Descriptive statistics

Means, standard deviations, and correlations between all the measures are presented in Table 1. First, as expected, PCSW and NCSW exhibited a positive correlation for each CSW domain. The correlations were generally moderate, ranging from 0.20 to 0.65 (*p*s < 0.05). Next, four one-dimensional CSW scores in each domain were obtained using the “depends” items (Depends CSW), “up” and “down” items (Positive and Negative CSW), all three types of items (All CSW), and Crocker et al. (2003) items (Crocker CSW). For each domain, the correlations between these four one-dimensional CSW scores were substantially high. The *r*s ranged in magnitude from 0.77 to 0.97 for others' approval, from 0.69 to 0.96 for appearance, from 0.78 to 0.97 for competition, from 0.78 to 0.95 for academics, from 0.78 to 0.97 for family support, from 0.73 to 0.97 for virtue, and from 0.86 to 0.99 for God's love. This indicates a high consistency among the four one-dimensional CSW indices. In addition, the Depends CSW, Positive and Negative CSW, All CSW, and Crocker CSW scores all positively correlated with PCSW and NCSW scores (*p*s < 0.05). Notably, most of the four one-dimensional CSW scores in the seven domains tended to have stronger correlations with NCSW and weaker correlations with PCSW, indicating that the one-dimensional CSW scores were more closely associated with NCSW (the Crocker CSW score was the only exception. It is possible that the numbers of Crocker et al.'s three types of items were not evenly distributed in some CSW domains. For example, three items of five items in the competition domain are “up” items, which may cause the Crocker CSW to possess a stronger correlation with PCSW than with NCSW).

### CFA on one-dimensional and two-dimensional models

As mention previously, employing items from heterogeneous CSW domains as measurement indicators under the same latent factor would lead to a poor goodness of fit when testing a CSW model. In addition, testing a correlated two-factor model for each single CSW domain could help estimate the correlation between PCSW and NCSW within each of the separate CSW domains. Thus, we used LISREL to conduct CFAs to test a correlated two-dimensional model and a competing one-dimensional model in each CSW domain, instead of conducting only one one-dimensional model and one two-dimensional model combining seven separate domains. Specifically, the one-dimensional model tested a single latent factor in each of the seven CSW domains with all up and down items in the domain that loaded onto it, reflecting a single dimension of contingent self-worth in the domain. The two-dimensional model tested two correlated latent factors (i.e., PCSW and NCSW), with all up items loading onto a PCSW latent factor and down items loading onto a NCSW latent factor, reflecting two dimensions of contingent self-worth in the domain. There were three up and three down items in the appearance, competition, academics, and family support domains; two up and three down items in the others' approval, virtue, and God's love domains. Evaluation of the fit of each model was based on multiple criteria. Because the Chi-Square statistic nearly always rejects the model when sample size is above 400 (our sample size was close to 400; Hair et al., 1998; Hooper et al., 2008), we primarily used four other fit indices to assess the degree to which the data fit the model: comparative fit index (CFI), non-normed fit index (NNFI), standardized root mean square residual (SRMR), and root mean square error of approximation (RMSEA). Following Hu and Bentler (1999) suggestion, SRMR values under 0.08 and RMSEA values under 0.06 indicate a good fit between the hypothesized model and the observed data. For the other fit indices, we used the commonly employed cutoff value of 0.90. The fit indices for the fourteen models (seven one- and seven two-dimensional models) are shown in Table 2.

**Table 2:** Goodness-of-Fit Summaries for Confirmatory Factor Models.

**Model**	**χ^2^**	***df***	**CFI**	**NNFI**	**SRMR**	**RMSEA**	**RMSEA 90% CI**
**OTHERS' APPROVAL CSW (*****N*** = **382)**							
One-dimensional model	240.35*	5	0.33	0.35	0.20	0.44	[0.40, 0.48]
Correlated two-dimensional model	22.44*	4	0.97	0.91	0.06	0.11	[0.07, 0.16]
**APPEARANCE CSW (*****N*** = **381)**							
One-dimensional model	461.29*	9	0.66	0.44	0.20	0.40	[0.37, 0.42]
Correlated two-dimensional model	36.30*	8	0.98	0.96	0.05	0.10	[0.06, 0.13]
**COMPETITION CSW (*****N*** = **382)**							
One-dimensional model	479.11*	9	0.59	0.32	0.19	0.39	[0.37, 0.42]
Correlated two-dimensional model	28.05*	8	0.98	0.97	0.05	0.08	[0.05, 0.11]
**ACADEMICS CSW (*****N*** = **384)**							
One-dimensional model	236.12*	9	0.81	0.69	0.11	0.28	[0.25, 0.30]
Correlated two-dimensional model	57.66*	8	0.96	0.92	0.06	0.12	[0.09, 0.15]
**FAMILY SUPPORT CSW (*****N*** = **380)**							
One-dimensional model	314.08*	9	0.72	0.53	0.15	0.32	[0.29, 0.35]
Correlated two-dimensional model	23.62*	8	0.99	0.97	0.05	0.07	[0.04, 0.11]
**VIRTUE CSW (*****N*** = **384)**							
One-dimensional model	53.14*	5	0.95	0.91	0.05	0.16	[0.12, 0.20]
Correlated two-dimensional model	12.77*	4	0.99	0.98	0.02	0.07	[0.03, 0.12]
**GOD'S LOVE CSW (*****N*** = **383)**							
One-dimensional model	410.97*	5	0.76	0.53	0.16	0.50	[0.46, 0.53]
Correlated two-dimensional model	56.58*	4	0.97	0.92	0.04	0.17	[0.13, 0.25]

CFI, comparative fit index; NNFI, non-normed fit index; SRMR, standardized root mean square residual; RMSEA, Root Mean Square Error of Approximation; RMSEA 90% CI, 90 Percent Confidence Interval for RMSEA;

* *p < 0.05*.

The results showed that most up and down items in the one-dimensional models in the seven CSW domains loaded significantly on their intended factors (i.e., a single CSW) with the only exception that a down item in the others' approval domain did not load significantly (standardized loading = 0.07, *p* > 0.05). Excluding this item, the standardized loadings ranged in magnitude from 0.21 to 0.88 for others' approval, from 0.35 to 0.88 for appearance, from 0.41 to 0.83 for competition, from 0.49 to 0.78 for academics, from 0.43 to 0.78 for family support, from 0.57 to 0.82 for virtue, and from 0.55 to 0.96 for God's love. Overall, the seven one-dimensional CSW models revealed relatively small values for fit indices (CFI and NNFI) and relatively big SRMRs and RMSEAs (see Table 2). Specifically, as to the six domains of others' approval, appearance, competition, academics, family support, and God's love, the values were all below 0.81 for the CFI, below 0.69 for the NNFI, above 0.11 for the SRMR, and above 0.28 for the RMSEA. These six one-dimensional CSW models did not provide an acceptably good fit to the data. In addition, as to the virtue domain, the values for CFI (0.95), NNFI (0.91), SRMR (0.05) were better than the cutoff values, only the value for RMSEA (0.16) was worse than the cutoff value. This indicates that the one-dimensional CSW model in the virtue domain seemed to be acceptable.

As to the two-dimensional CSW models, the results revealed that all up and down items in the seven domains loaded significantly on two correlated intended factors (i.e., PCSW and NCSW), with standardized loadings ranging in magnitude from 0.62 to 0.92 for others' approval, from 0.73 to 0.91 for appearance, from 0.66 to 0.90 for competition, from 0.62 to 0.85 for academics, from 0.62 to 0.86 for family support, from 0.69 to 0.85 for virtue, and from 0.83 to 0.97 for God's love. The correlations between PCSW and NCSW in the seven domains were mostly moderate, Φs = 0.23, 0.39, 0.35, 0.57, 0.42, 0.76, 0.69, *p*s < 0.05. Overall, the seven correlated two-dimensional models tended to reveal relatively big values for fit indices (CFI and NNFI) and relatively small SRMRs and RMSEAs compared with the seven one-dimensional models. Specifically, as to the seven CSW domains of others' approval, appearance, competition, academics, family support, virtue, and God's love, although the values for RMSEA (ranging from 0.07 to 0.17) were somewhat worse than the cutoff value (0.06), the values for CFI (all above 0.96), NNFI (all above 0.91), SRMR (all below 0.06) were better than the cutoff values. Thus, the two-dimensional CSW models in these seven domains seemed to be acceptable. Furthermore, χ^2^ difference tests were used to compare the model fit between the seven correlated two-dimensional models and seven one-dimensional models. The results indicated that the seven correlated two-dimensional models fit significantly better than the seven one-dimensional models separately, Δχ^2^s (1) = 217.91, 424.99, 451.06, 178.52, 290.16, 40.37, 354.39, *p*s < 0.05. This suggests that CSW in the seven domains was better seen as comprising two distinct factors (PCSW and NCSW) with a positive correlation between them, instead of being considered as a one-dimensional construct.

### The association between CSW and depression

Schwinger et al. (2017) indicated that the domain-specific CSW scores are more important than global scores of CSW for the prediction of depressive symptoms. In addition, to more clearly compare the proportions of variance in depressive symptoms explained by one-dimensional and two-dimensional CSW, we also conducted the regression analyses separately in each CSW domain. Specifically, we conducted five regression analyses in each CSW domain, including one two-factor model (PCSW and NCSW entered as predictor variables and depressive symptoms entered as the criterion variable) and four one-factor models (Positive and Negative CSW, Depends CSW, All CSW, and Crocker CSW entered as predictor variables separately and depressive symptoms as the criterion variable). In order to reduce the risk of Type I error and maintain the 95% confidence, we used the Bonferroni correction (Moiseev, 2017) to set the *p*-value as 0.01(0.05/5 = 0.01). Table 3 shows the results of the regression analyses.

**Table 3:** Regression Results of the Two- and One-Dimensional CSW on Depression in Seven Domains.

	**MODEL**		**Estimate**	***SE***	***df***	**β**	***t***	***R*^2^**
Others' Approval	Two factors	PCSW	−0.10	0.02	376	−0.20	−4.08*	0.15
		NCSW	0.14	0.02		0.37	7.61*
	One Factor	Depends CSW	0.09	0.02	380	0.24	4.88*	0.06
		Positive and Negative CSW	0.12	0.03	380	0.22	4.43*	0.05
		All CSW	0.12	0.02	380	0.25	4.96*	0.06
		Crocker CSW	0.10	0.02	380	0.27	5.48*	0.07
Appearance	Two factors	PCSW	−0.13	0.02	375	−0.28	−5.69*	0.19
		NCSW	0.17	0.02		0.44	8.82*
	One Factor	Depends CSW	0.07	0.02	379	0.17	3.40*	0.03
		Positive and Negative CSW	0.08	0.03	376	0.15	2.98*	0.02
		All CSW	0.09	0.03	375	0.17	3.41*	0.03
		Crocker CSW	0.07	0.02	380	0.16	3.14*	0.03
Competition	Two factors	PCSW	−0.10	0.03	376	−0.20	−3.96*	0.13
		NCSW	0.14	0.02		0.37	7.35*
	One factor	Depends CSW	0.06	0.02	380	0.14	2.69*	0.02
		Positive and Negative CSW	0.09	0.03	377	0.18	3.48*	0.03
		All CSW	0.09	0.03	377	0.17	3.37*	0.03
		Crocker CSW	−0.01	0.03	380	−0.02	−0.29	0.00
Academics	Two factors	PCSW	−0.13	0.00	378	−0.26	−4.68*	0.10
		NCSW	0.14	0.02		0.35	6.33*
	One factor	Depends CSW	0.07	0.02	376	0.17	3.23*	0.03
		Positive and Negative CSW	0.05	0.03	379	0.10	1.99	0.01
		All CSW	0.07	0.03	375	0.14	2.69*	0.02
		Crocker CSW	0.06	0.03	380	0.12	2.38	0.02
Family Support	Two factors	PCSW	−0.12	0.03	374	−0.20	−4.65*	0.08
		NCSW	0.10	0.02		0.26	4.86*
	One factor	Depends CSW	0.00	0.02	378	−0.01	−0.17	0.00
		Positive and Negative CSW	0.02	0.03	375	0.04	0.71	0.00
		All CSW	0.01	0.03	374	0.02	0.43	0.00
		Crocker CSW	−0.02	0.03	379	−0.04	−0.84	0.00
Virtue	Two factors	PCSW	−0.13	0.03	379	−0.28	−4.53*	0.06
		NCSW	0.10	0.03		0.24	3.90*
	One factor	Depends CSW	−0.02	0.02	379	−0.04	−0.79	0.00
		Positive and Negative CSW	0.00	0.02	380	−0.01	−0.14	0.00
		All CSW	−0.01	0.03	380	−0.02	−0.42	0.00
		Crocker CSW	0.01	0.02	380	0.01	0.19	0.00
God's Love	Two factors	PCSW	−0.06	0.02	377	−0.20	−2.95*	0.03
		NCSW	0.06	0.02		0.18	2.72*
	One factor	Depends CSW	0.01	0.02	378	0.03	0.64	0.00
		Positive and Negative CSW	0.00	0.02	380	−0.00	−0.08	0.00
		All CSW	0.00	0.02	379	0.01	0.15	0.00
		Crocker CSW	−0.01	0.02	380	−0.03	−0.48	0.00

PCSW, Positive CSW obtained from “up” items; NCSW, Negative CSW obtained from “down” items; Depends CSW, One-dimensional CSW obtained from “depends” items; Positive and Negative CSW, One-dimensional CSW obtained from “up” and “down” items; All CSW, One-dimensional CSW obtained from “up”, “down”, and “depends” items; Crocker CSW, One-dimensional CSW obtained from Crocker et al. (2003) items;

* *p < 0.01*.

Regarding the relationship between one-dimensional CSW and depressive symptoms, the results showed that in the domains of others' approval and appearance, the four one-dimensional CSW indices (Positive and Negative CSW, Depends CSW, All CSW, and Crocker CSW) were all positively associated with depressive symptoms (βs ranged from 0.22 to 0.27 for others' approval, from 0.15 to 0.17 for appearance, *p*s < 0.01). For the competition domain, the Positive and Negative CSW, Depends CSW and All CSW were also related to greater depressive symptoms (βs ranged from 0.14 to 0.18, *p*s < 0.01). However, the relationship between Crocker CSW and depressive symptoms was not significant (*p* > 0.01). For the academics domain, the Depends CSW and All CSW were also related to greater depressive symptoms (βs were 0.17 and 0.14, *p*s < 0.01). However, the relationship between depressive symptoms and Crocker CSW or Positive and Negative CSW was not significant (*p*s > 0.01). In addition, the four one-dimensional CSW indices in the domains of family support, virtue, and God's love did not correlate with depressive symptoms (βs < 0.04, *p*s > 0.01). These results generally indicate that people whose self-worth easily fluctuates with positive or negative events in the more external CSW domains (i.e., others' approval, appearance, competition, and academics) are more likely to exhibit depressive symptoms. However, in terms of internal CSW domains (i.e., virtue and God's love), whether self-worth is easily influenced by positive or negative events is not related to depressive symptoms.

Next, as to the two-factor model, the results showed that when PCSW and NCSW entered simultaneously in the regression equations, PCSW was associated with less depressive symptoms in each of the seven CSW domains (βs ranged from −0.20 to −0.28, *p*s < 0.01); NCSW was associated with greater depressive symptoms in each domain (βs ranged from 0.18 to 0.44, *p*s < 0.01). This indicates that people whose self-worth easily increases because of positive events are less likely to exhibit depressive symptoms, and people whose self-worth easily decreases because of negative events tend to exhibit more depressive symptoms. In addition, PCSW and NCSW jointly accounted for 15, 19, 13, 10, 8, 6, and 3% of the variance in depressive symptoms in seven domains, which were all relatively higher than the proportions of the variance explained by one-dimensional CSW in seven domains (the proportions were below 7, 3, 3, 3, 1, 1, and 1, respectively).

We further examined whether the explained variance in depressive symptoms could be increased by adding any of the four one-dimensional CSW scores into the two-factor regression models (PCSW and NCSW were already two predictor variables) in seven CSW domains. The results showed that adding one-dimensional CSW indices into the two-factor regression equations in seven CSW domains did not increase the explained variance in depressive symptoms (Δ*R*^2^s < 0.011, *p*s > 0.01), indicating that the proportion of variance in depressive symptoms accounted for by two-dimensional CSW have contained the proportion of variance explained by one-dimensional CSW in each CSW domain. These results indicate that relative to using one-dimensional CSW as a predictor of depressive symptoms, using PCSW and NCSW as predictor variables simultaneously would largely increase the proportion of variance in depressive symptoms accounted for, although the items used to measure PCSW and NCSW were just the same with the items measuring Positive and Negative CSW or similar to those measuring the other three one-dimensional CSW indices (i.e., Depends CSW, All CSW, and Crocker CSW).

In addition, some studies indicated that CSW did not associate with depressive symptoms when controlling for self-esteem (e.g., Wouters et al., 2013b; Sowislo et al., 2014). To test this, we further conducted all of the above-mentioned one-factor and two-factor regressions after controlling for global self-esteem by using the hierarchical regression analyses. In all of the regression models, we first entered global self-esteem at Step 1, which showed a significant positive effect on depressive symptoms (β = 0.65, *p* < 0.001). At Step 2, as to the one-factor regression models, all of the four one-dimensional CSW scores in each CSW domain did not increase the proportion of variance in depressive symptoms accounted for (Δ*R*^2^s < 0.003, *p*s > 0.05). The results were consistent with the findings of previous studies. However, as to the two-factor regression models, although PCSW and NCSW of others' approval, academics, competition, family support, and God's love domains also did not associate with depressive symptoms after controlling for global self-esteem (Δ*R*^2^s < 0.003, *p*s > 0.05), two-dimensional CSW of appearance and virtue domains increased the proportion of variance in depressive symptoms accounted for (Δ*R*^2^s > 1%, *p*s = 0.022, 0.05). NCSW of appearance showed a positive effect (β = 0.13, *p* = 0.007) and PCSW of virtue showed a negative effect (β = −0.12, *p* = 0.017) on depressive symptoms after controlling for global self-esteem. The results indicated that after dividing CSW into two dimensions of PCSW and NCSW, certain domains of CSW were associated with depressive symptoms even when controlling for global self-esteem.

## Discussion

Previous studies assumed people's CSW on positive and negative events as the same construct and tended to combine various types of items to create a one-dimensional CSW index. Although one-dimensional CSW did associate with depressive symptoms in the expected ways, the effect seemed to be generally weak. We considered this is at least partially related to a basic but critical problem regarding the CSW theory and measurement. That is, people's CSW on positive and negative events are possibly two distinctive dimensions. Below we interpreted the findings and discussed the implications.

### PCSW and NCSW as two distinctive dimensions

Overall, the results of this study clearly provided support that people's CSW on positive and negative events are better seen as two distinctive dimensions instead of a one-dimensional CSW construct. First, the results revealed that the correlations between PCSW and NCSW were generally moderate in the seven CSW domains, indicating that PCSW and NCSW are not identical constructs for most CSW domains. More importantly, when we focused on a single CSW domain and adopted PCSW and NCSW as two latent factors, the results of CFA indicated that this two-dimensional model was superior at explaining the observed data for all seven CSW domains than the one-dimensional model, which adopted a one-dimensional CSW construct as a latent factor. Thus, PCSW and NCSW are better seen as two distinctive dimensions. This finding helps enhance the understanding of individual differences in people's CSW. That is, regardless of CSW domains, possessing a vulnerable self-esteem (easily lowered by negative events) for a person does not necessarily mean that this person would possess a self-esteem easily increased by positive events.

### Two-dimensional CSW increases explanatory power in explaining depression

The results of the regression analyses showed that the direction and magnitude of the relationships between the four one-dimensional CSW indices and depressive symptoms were similar. In most external CSW domains (i.e., others' approval, appearance, competition, academics), each of the four one-dimensional CSW indices was associated with greater depressive symptoms. However, in the internal CSW domains, each of the four one-dimensional CSW indices was unrelated to depression. This is consistent with the findings of previous studies. That is, internal CSW is relatively healthy which would exhibit a low negative correlation or no correlation with depression-related concepts (e.g., Crocker et al., 2003; Sargent et al., 2006). However, when people tend to emphasize more on the external events and their self-worth easily fluctuates because of these positive and negative events (external CSW), such self-esteem instability would lead to depression (e.g., Crocker and Park, 2004; Crocker and Knight, 2005; Sanchez and Crocker, 2005; Burwell and Shirk, 2006; Sargent et al., 2006; Cambron et al., 2010).

Next, the regression analyses also showed that PCSW and NCSW exhibited independent effects for depression. In each of the seven CSW domains, PCSW was associated with less depressive symptoms and NCSW was associated with greater depressive symptoms. This is consistent with our predictions. According to the previous studies (e.g., Kernis, 2003; Fredrickson and Losada, 2005; Schultz and Heimberg, 2008; Gotlib and Joormann, 2010; vanDellen et al., 2011), we proposed that people whose self-worth is easily increased by positive events in various domains are less likely to exhibit depressive symptoms, and those whose self-worth is easily decreased by negative events tend to show more depressive tendencies. More importantly, in each of the seven CSW domains, when PCSW and NCSW entered simultaneously in the regression equation, the variance of depression explained by PCSW and NCSW had largely increased compared with using one-dimensional CSW as a predictor of depression. This indicates that dividing CSW into two dimensions of PCSW and NCSW may largely increase the utilization value of CSW in explaining depression. This finding may also help explain why the previous studies have found a weak relationship between CSW and depression. For example, some studies indicated that CSW did not associate with depressive symptoms when controlling for self-esteem (e.g., Wouters et al., 2013b; Sowislo et al., 2014). However, our studies showed that after dividing CSW into two dimensions of PCSW and NCSW, certain domains of CSW did associate with depressive symptoms, even when controlling for global self-esteem. In brief, previous researchers considered CSW on positive and negative events as the same construct instead of distinguishing it into two differing dimensions. This may lead to underestimating the relationship between CSW and mental health or even other variables.

These results have some important theoretical and practical implications. First, past studies have indicated that self-worth easily fluctuates because of external events (i.e., high self-esteem instability) may harm people and easily induce mental health problems (e.g., depression) (e.g., Crocker et al., 2003; Crocker and Park, 2004; Crocker and Knight, 2005; Cambron et al., 2010; Wouters et al., 2013a). Thus, the external CSW, which leads to self-esteem instability, is a risk factor to depression. However, this study showed that when self-worth easily fluctuates (reduces) during negative events (i.e., high NCSW), this would be bad for people's mental health. However, when self-worth easily fluctuates (increases) because of positive events (i.e., high PCSW), this kind of self-esteem instability may be not related to mental health problems. In brief, maybe only NCSW is a risk factor to depression and PCSW may be not.

In addition, the “up,” “down,” and “depends” items were used to measure CSW in the previous studies. The current study calculated four types of one-dimensional CSW scores according to these three types of items and showed that the one-dimensional CSW scores tended to exhibit relatively high correlation with “down” items and relatively low correlation with “up” items (see Table 1). That is, the one-dimensional CSW scores seemed to be more closely associated with NCSW. This may help explain why the one-dimensional CSW scores in most external domains related with depression in the same (positive) direction with NCSW. However, notice that the relationships between the one-dimensional CSW scores and depressive symptoms were much lower than those between NCSW and depressive symptoms in all CSW domains (see Table 2). Thus, using combined one-dimensional CSW scores to explain depression not only may neglect the explanatory power of PCSW but also may reduce that of NCSW. Under such circumstance, we recommend future studies to use only “up” and “down” items to measure CSW and conduct independent scoring for PCSW and NCSW. When PCSW and NCSW enter simultaneously in the regression equation, the explanatory power of CSW for relevant constructs may largely improve.

### Limitations and future directions

The current study has several limitations related to issues with research potential. First, because no variables in our study were manipulated and it is a cross-section design, we are unable to draw inferences about the causal directions between CSW and depression. More studies using other research methods (e.g., experiment method or longitudinal design) are needed to examine the causal relationships between these constructs and clarify the possible underlying processes. Next, because the participants of this study were Taiwanese university students, the results may only be applicable to participants from an Asian cultural background. That is, cultural differences among the research samples may have influenced this study's results. We recommend that future studies include participants from various cultures, testing whether the findings in the present study resulted from cross-cultural differences. Finally, the results showed that distinguishing CSW on positive and negative events into PCSW and NCSW would increase the proportion of variance in depression accounted for by CSW. Future studies can continue to explore whether viewing CSW as a two-dimensional perspective, compared to a one-dimensional perspective, would help increase the explanatory power of CSW for constructs that were also widely investigated in previous CSW studies, such as motivation and self-regulation behaviors (Crocker et al., 2006) and alcohol and drug use behaviors (Crocker, 2002; Luhtanen and Crocker, 2005).

## Ethics statement

This study was carried out in accordance with the recommendations of Institutional and National 535 Guidelines and with the Declaration of Helsinki (2013) with written informed consent from all subjects. All subjects gave written informed consent in accordance with the Declaration of Helsinki.

## Author contributions

C-HL and P-SH study conceptualization, data collection, data analysis, interpretation of results and write-up.

### Conflict of interest statement

The authors declare that the research was conducted in the absence of any commercial or financial relationships that could be construed as a potential conflict of interest.

## Appendix

Added items in the seven CSW domains.

**Table:**

**Domain**	**Items**
Others' Approval	-When others have positive opinions about me, my self-esteem increases (up item). -If others value who I am, I feel great about myself (up item). -When others have negative opinions about me, I feel bad about myself (down item).
Appearance	-My self-esteem is enhanced when I find my physical appearance look good (up item). -My sense of self-worth increases when I think some parts of physical appearance look nice (up item). -I feel bad about myself when I think some parts of my physical appearance look unattractive (down item). -When I think of myself as unattractive, I have bad feelings about myself (down item).
Competition	-The results of my competitions with others do not affect the degree of my self-esteem (depends item).^*^ -When I am not as competitive as others, I feel bad about myself (down item). -When I perform not as well as others on a task or a skill, my self-esteem decreases (down item). -My self-evaluation is not affected when I am compared down in a competition (down item).^*^
Academics	-My self-esteem increases when I do well academically (up item). -My academic performance affects the degree of my self-esteem (depends item). -My self-esteem decreases when I do poorly academically (down item). -My sense of self-worth is not affected when I have poor academic performance (down item).^*^
Family Support	-My self-esteem increases when my family members respect me (up item). -My relationship with family members affects the degree of my self-esteem (depends item). -I feel bad about myself when I have poor relationship with my family members (down item). -My sense of self-worth is lowered when my family members see me negatively (down item).
Virtue	-I feel good about myself when what my doings are consistent with my moral principles (up item). -Whether my actions are based on my moral standards affect my self-evaluation (depends item). -Whether my actions are consistent with moral principles is unrelated with the degree of my self-esteem (depends item).^*^
God's Love	-I feel bad about myself when I feel God do not love me (down item). -The degree of my self-esteem is affected by whether I feel God' love toward me (depends item).

*Items marked with “*” are reverse coded item*.
